# Active Aging and the Mattheus Effect

**DOI:** 10.1093/geroni/igab046.1877

**Published:** 2021-12-17

**Authors:** Per Jensen

**Affiliations:** Aalborg University, Aalborg, Nordjylland, Denmark

## Abstract

While active ageing has been discursivized in international organizations and among researchers as a major means to combat the challenges of demographic ageing, this study aims to make a critical-theoretical and empirical assessment of the active ageing concept. It falls into three parts, the first showing how the conceptual framework of active ageing is undertheorized, lacks conceptual and analytical clarity, and that the theoretical framework does not hold clear ideas regarding the factors conditioning active ageing. The second part investigates the main patterns and structuring mechanisms of active ageing in an outcome perspective using Danish data subject to a correspondence analysis. Here, a Matthew Effect of accumulated advantage is found; that is, older adults who are blessed in one sphere of life are also blessed in others, and such inequalities in old age are the outcomes of social life biographies (i.e., cumulative advantages/disadvantages over the life course). Although nursed by the political system, EU ideas about active ageing are only weakly translated into policies and programs. Part three discusses some of the reasons for this, one obviously being that active ageing is elusive and lacks well-defined cause-and-effect descriptions. Another reason is that the concept has been developed in global elite networks that are quite distant from policymakers; at least in a decentralized political system like the Danish welfare state.

